# Novel evidence that an alternative complement cascade pathway is involved in optimal mobilization of hematopoietic stem/progenitor cells in Nlrp3 inflammasome-dependent manner

**DOI:** 10.1038/s41375-019-0530-9

**Published:** 2019-07-26

**Authors:** Mateusz Adamiak, Anna M. Lenkiewicz, Monika Cymer, Magda Kucia, Janina Ratajczak, Mariusz Z. Ratajczak

**Affiliations:** 1 Stem Cell Institute at James Graham Brown Cancer Center, University of Louisville, Louisville, KY USA; 2 Department of Regenerative Medicine, Warsaw Medical University, Warsaw, Poland

**Keywords:** Haematopoietic stem cells, Oncogenesis

## To the Editor:

The mobilization of stem cells is still not well understood, despite the fact that this process is important for understanding the response of the organism to inflammation, tissue and organ injury, and pharmacological mobilization as a clinical procedure to harvest hematopoietic stem/progenitor cells (HSPCs) for hematopoietic transplantation [1, 2]. Currently, several mediators and cells have been proposed as playing a pivotal role in this phenomenon. Specifically, evidence has accumulated that this process is orchestrated by ancient evolutionary mechanisms mediated by innate immunity. All cell types belonging to the innate immunity network (granulocytes, monocytes, and dendritic cells) as well as the complement cascade (ComC) here play a crucial role [2, 3].

The ComC is a part of the innate immune system that defends against infections. However, evidence has accumulated that it has several other effects, including involvement in organ development, tissue regeneration, and stem cell trafficking [4]. Our group proposed that the release of HSPCs from bone marrow (BM) into peripheral blood (PB) is orchestrated by the induction of “sterile inflammation” in the BM microenvironment in response to infection, tissue/organ injury, strenuous exercise, or the administration of pro-mobilizing drugs [5, 6], such as cytokine granulocyte colony-stimulating factor (G-CSF) or AMD3100, a small-molecule inhibitor of the chemokine receptor CXCR4. G-CSF-induced or AMD3100-induced pharmacological mobilization of HSPCs is a means of obtaining these cells in the clinic for hematopoietic transplantation.

The ComC consists of zymogen proteins that become activated in a cascade-mediated manner by the (i) classical, (ii) mannan-binding lectin (MBL), or (iii) alternative pathways [4]. Our previous work demonstrated that the MBL but not the classical pathway plays a pivotal role in pharmacological mobilization with G-CSF or AMD3100 [7]. Specifically, mice deficient in the C1q protein, which initiates classical ComC activation, are good G-CSF and AMD3100 mobilizers [7, 8], in contrast to animals that are deficient in MBL or mannan-activated serum protease 2 (MASP-1) [7]. However, since MBL-KO and MASP-1-KO mice still mobilize some HSPCs in response to G-CSF and AMD3100, here we asked whether the alternative pathway of ComC activation plays a compensatory role in this process. To address this question, we performed mobilization studies in factor B (FB)-deficient (FB-KO) mice. FB is a serine protease that is required for activation of the alternative ComC pathway. In contrast to the other two pathways, the alternative pathway is not triggered by antibodies or specific structures on the surface of microorganisms. Instead, it is activated by the spontaneous hydrolysis of C3 (the third component of the ComC and the most abundant complement protein present in blood plasma) [9].

Furthermore, supporting the important role of innate immunity in mobilization, we recently demonstrated involvement of the Nlrp3 inflammasome complex in the induction of “sterile” inflammation in BM, which triggers the mobilization of HSPCs [10, 11]. Based on this and our previous data we become interested in a potential role of alterative pathway of ComC activation in mobilization of HSPCs. Pathogen-free, 4–6-week-old FB-deficient mice were mobilized with G-CSF (Amgen, Thousand Oaks, CA, USA) for 3 days at 100 μg/kg per day by subcutaneous injection (SC) with AMD3100 (Sigma-Aldrich, St. Louis, MO, USA) for 1 day at 5 mg/kg by intraperitoneal injection (IP). Following mobilization, we measured (i) the total number of white blood cells (WBCs), (ii) the number of Sca-1^+^c-kit^+^lineage^–^ (SKL) cells in PB, and (iii) the number of circulating clonogenic colony-forming unit granulocyte/macrophage (CFU-GM) progenitors. In parallel, we evaluated by RQ-PCR changes in expression of genes involved in Nlrp3 inflammasome activation and by ELISA plasma level of C5 cleavage fragment C5a ELISA, and Nlrp3 inflammasome released Hmgb1, IL-1β, and IL-18 [12].

**Fig. 1:**
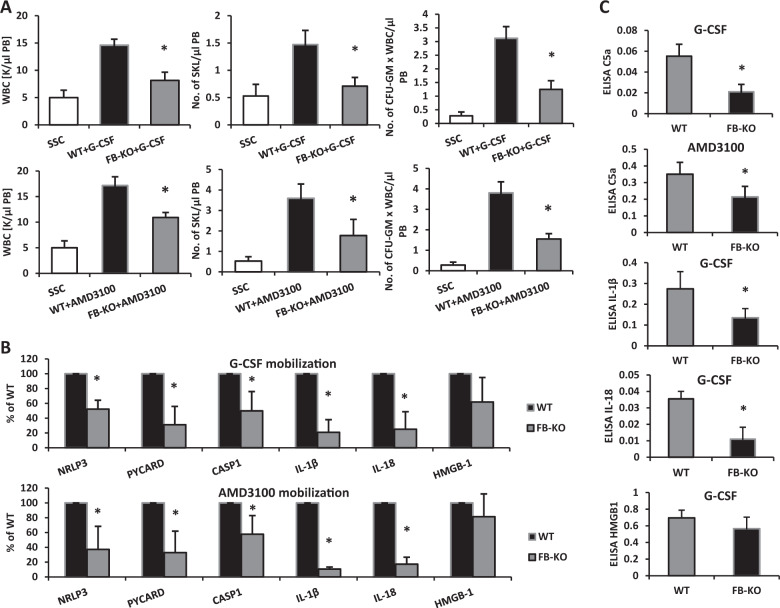
Panel (**a**) FB-KO mice are poor HSPC mobilizers. For mobilization studies mice (six animals per group) were mobilized with G-CSF (Amgen, Thousand Oaks, CA, USA) for 3 days (short mobilization) at 100 μg/kg per day by subcutaneous injection (SC) with AMD3100 (Sigma-Aldrich, St. Louis, MO, USA) for 1 day at 5 mg/kg by intraperitoneal injection (IP). At 6 h after the last G-CSF injection, 1 h after AMD3100 injection, the mice were bled from the retro-orbital plexus for WBCs analysis, and PB was obtained from the vena cava (with a 25-gauge needle and 1-ml syringe containing 250 U heparin) for SKL (Sca-1^+^/c-kit^+^/Lin^−^) cells, CFU-GM clonogenic progenitors and ELISA analysis. Mononuclear cells (MNCs) were obtained by hypotonic lysis of RBCs in BD Pharm Lyse buffer (BD Biosciences) as described. Results from two independent experiments are pooled together. *p ≤ 0.05. Panels (**b**) and (**c**) Impaired activation of the Nlrp3 inflammasome in mobilized FB-KO mice. Panel (**b**) The effect of G-CSF on Nlrp3 inflammasome activation was evaluated at the mRNA level in PB. The expression of NLRP3, ASC (Pycard), CASP1, IL-1β, IL-18, HMGB1, S100a9, AIM2, GSDMD, NLRP1a, and NLRP1b mRNAs in PB after G-CSF and AMD3100 mobilization was measured by qRT-PCR. Sequences of primers and amplification parameters employed for these studies are shown in ref. [11]. Panel (**c**) The effect of G-CSF on Nlrp3 inflammasome activation was evaluated at the protein level by ELISA in PB plasma. The C5a, IL-1β, IL-18, and Hmgb-1 levels were measured using enzyme-linked immunosorbent assay (ELISA) kits (C5a, Cloud-Clone cat. no. SEA388Mu; IL-1β, Cloud-Clone cat. no. SEA563Mu; IL-18, Affymetrix eBioscience cat. no. BMS618/3; HMGB-1, Cloud-Clone cat. no. SEA399Mu), according to the manufacuterers’ instructions. Results (absorbance) are presented as the percentage of control. Results from two independent experiments are pooled together. *p ≤ 0.01

We found that under steady-state conditions FB-KO mice have normal PB cell counts, red blood cell parameters, numbers of BM-residing and PB-circulating HSPCs, and numbers of clonogenic progenitors in BM compared with WT animals (Supplementary Fig. 1). These animals were subsequently employed along with sex-matched and age-matched syngeneic controls in our pharmacological mobilization induced by G-CSF or AMD3100 (Fig. 1a). We noticed that these animals turned out to be poor stem cell mobilizers. The numbers of mobilized WBCs, SKL cells, and CFU-GM clonogenic progenitors were significantly lower in FB-KO mice than in control wild type (WT) mice. Moreover, we found that, in addition to HSPCs, FB-KO animals displayed decreased mobilization of other types of BM-residing stem/progenitor cells, including mesenchymal stroma cells (MSCs) and endothelial progenitor cells (EPCs), as well as a rare population of stem cells, very small embryonic like stem cells (VSELs) (Supplementary Fig. 2).

This result shows that FB-mediated activation of the alternative pathway of ComC activation is important for optimal release of all types of BM-residing stem cells into PB.

In our recent paper as mentioned above, we provided evidence for a crucial role for Nlrp3 inflammasome activation in the pharmacological mobilization of HSPCs [10, 11]. Based on this evidence we analyzed the expression of genes involved in the inflammasome complex with respect to mRNA (Fig. 1b) and protein (Fig. 1c). We found a decrease in expression of mRNA for Nlrp3, ASC (Pycard), caspase 1, Hmgb1, IL-1β, and IL-18 in mRNA isolated from BM and PB MNCs from FB-KO mice. This defect in activation of the ComC and the Nlrp3 inflammasome was subsequently confirmed at the protein level, as we demonstrated a decrease in markers of Nlrp3 inflammasome activation circulating in PB, such as IL-1β, IL-18, and Hmgb1 [12], in parallel with a decrease in level of the distal cleavage fragment of ComC activation C5a [11].

**Fig. 2:**
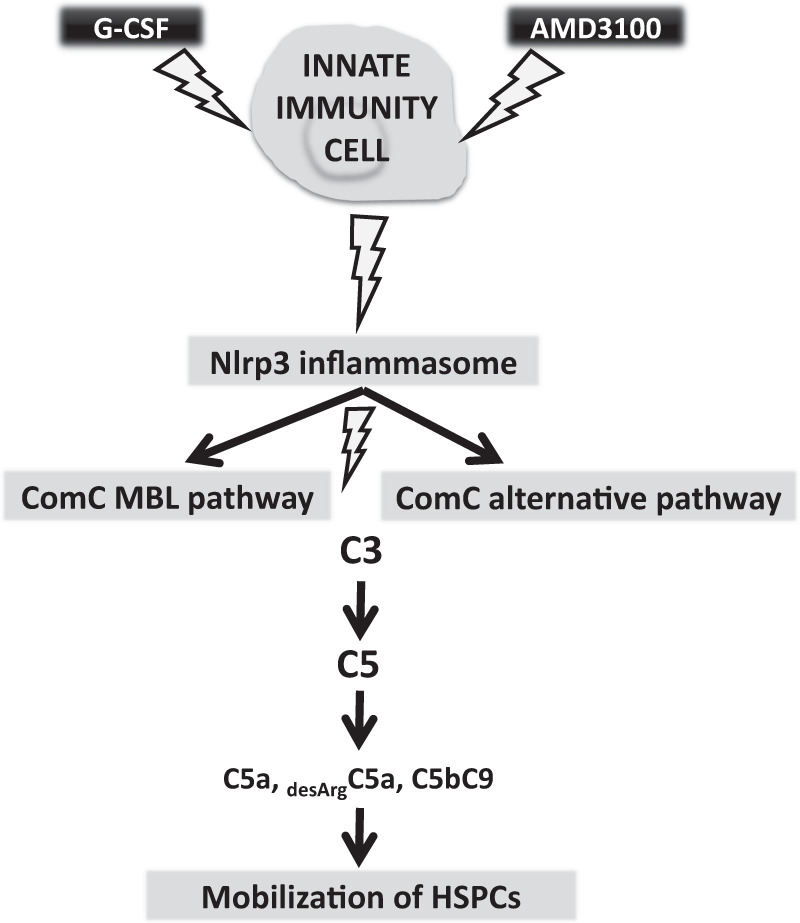
Alternative pathway of ComC activation supports MBL pathway of ComC activation in Nlrp3 inflammasome-dependent manner. Pharmacological mobilization of HSPCs after administration of G-CSF or AMD3100 activates Nlrp3 inflammasome in innate immunity cells what leads to activation of mannan-binding lectin (MBL) and alternative pathway of ComC activation

During the mobilization process both the MBL and the alternative pathways of ComC activation lead to generation of the C5 cleavage fragments, the anaphylatoxins C5a and _desArg_C5a, as well as the soluble C5b-C9 membrane attack complex (MAC), which we found to promote egress of stem cells from BM into PB [13]. Our results show redundancy between the MBL and alternative pathways in this process (Fig. 2). Based on this finding, it would be interesting to see whether simultaneous perturbation of the MBL and alternative pathways has a more profound effect than a single perturbation of one pathway or the other.

As we have recently demonstrated, adenosine triphosphate (ATP) that is released from innate immunity cells after administration of pro-mobilizing agents is an important trigger for Nlrp3 inflammasome activation [10, 11]. Several mediators released from innate immunity cells after Nlrp3 inflammasome activation, such as IL-1β, IL-18, and members of the family of danger-associated molecular pattern molecules (DAMPs), including high-mobility group box 1 protein (Hmbg1) and S1000a9 protein, lead to activation of the ComC [10, 11]. This activation occurs via MASP-1, which also activates in parallel the coagulation cascade (CoaC) in the BM microenvironment and as reported CoaC supports mobilization process [14].

The crucial role of the ComC in egress of HSPCs from BM has been demonstrated in C5-KO mice [2], which do not generate the soluble mediators C5a, _desArg_C5, and C5b-C9 in the distal part of ComC activation. C5 and _desArg_C5a activated in the BM microenvironment potentiate Nlrp3 inflammasome activation and in PB provide a chemotactic gradient for granulocytes and monocytes to egress from BM. These innate immunity cells are enriched in proteolytic enzymes, including metalloproteinase 9 (MMP-9), and play an important role in permeabilization of the BM–PB endothelial barrier in BM sinusoids. As a consequence, HSPCs chemoatttacted by the S1P gradient in PB follow in the path of these cells to egress from the BM [15]. In the process of permeabilization of the endothelial barrier in BM, the anaphylatoxin C5a and its receptor C5aR, which is expressed on the surface of endothelial cells, also play a direct role [13]. Another C5 cleavage product, C5b, which leads to generation of MAC (C5b-C9), may additionally increase the chemotactic level of sphingosine-1 phosphate (S1P) for HSPCs in blood plasma [15] by triggering its release from erythrocytes and platelets.

What is also important is that, besides ATP, both C5a and C5bC9 are known activators of the Nlrp3 inflammasome in innate immunity cells [10]. In the present work we observed that, in contrast to WT animals, the Nlrp3 inflammasome is less efficiently activated in the BM of FB-KO animals in response to mobilizing drugs. This was expected, and accordingly we observed lower expression of mRNAs for Nlrp3 inflammasome components, as well as lower levels of IL-1β and IL-18 (which are markers of Nlrp3 inflammasome activation) released into PB in FB-KO mice.

In conclusion, we have demonstrated for the first time that the alternative pathway of ComC activation is involved in mobilization of hematopoietic and non-hematopoietic stem cells from BM into PB. We also demonstrated that this process occurs in an Nlrp3 inflammasome-mediated manner, as FB-KO mice activate this inflammasome components poorly. Moreover, since mobilization was not completely inhibited in FB-KO mice, as in our previous studies in MBL-KO animals, this result suggests a compensatory and redundant role for the MBL and alternative pathways in the mobilization process.

## Supplementary information

Legends for Supplementary Figures

Supplementary Figure 1A

Supplementary Figure 1 B and C

Supplementary Figure 2A

Supplementary Figure 2B
