# Supplementary material for: Novel evidence that an alternative complement cascade pathway is involved in optimal mobilization of hematopoietic stem/progenitor cells in Nlrp3 inflammasome-dependent manner
Source: Leukemia. 2019 Jul 26;33(12):2967–70. doi: 10.1038/s41375-019-0530-9 (PMC8076004; doi:10.1038/s41375-019-0530-9)
Supplement: Supplementary file 1 — Legends for Supplementary Figures [file 41375_2019_530_MOESM1_ESM.docx]

**Legends for Supplementary Figures**

**Supplementary Figure 1.**

Hematological parameters in FB-KO compared with WT mice. Panel A. Peripheral blood (PB) parameters were evaluated using a HemaVet 950FS analyzer. There was no significant difference in the numbers of white blood cells (WBCs), neutrophils (NEs), lymphocytes (LYs), monocytes (MOs), and platelets (PLTs) or in the numbers of RBCs, hemoglobin content (HB), hematocrit (HCT), mean volume of erythrocytes (MCV), mean content of hemoglobin (MCH), mean concentration of hemoglobin in erythrocytes (MCHC), and red cell distribution width (RDW). Panel B. Under steady-state conditions, there were also no significant differences between FB-KO and WT mice in the numbers of Lin−/Sca-1+/c-kit+ (SKL) cells or HSCs in BM and circulating in PB. Panel C. Bone marrow (BM) of mice was also isolated and evaluated for the numbers of colony-forming unit granulocyte/macrophage (CFU-GM) and erythroid progenitor cells (BFU-E) in in vitro assays. FB-KO–/– and control mice had similar numbers of CFU-GM and BFU-E clonogenic progenitors. The data represent an average of at least 8 mice tested per experimental group (*p ≤ 0.05).

**Supplementary Figure 2.**

FB-KO mice are poor MSC, EPC, and VSEL mobilizers. To assess the mobilization of other stem cells residing in BM, PB mononuclear cells were isolated from WT mice after treatment for 3 days with G-CSF (Panel A) or 1 hour after AMD3100 administration (Panel B). The numbers of Lin–/CD45–/CD31–/CD90+ (MSCs), Lin–/CD45–/CD31+ (EPCs), and Sca-1+/Lin–/CD45– (VSELs) were evaluated by FACS. Results from two independent experiments are pooled together. *p≤0.05
