# Supplementary material for: Novel evidence that an alternative complement cascade pathway is involved in optimal mobilization of hematopoietic stem/progenitor cells in Nlrp3 inflammasome-dependent manner
Source: Leukemia. 2019 Jul 26;33(12):2967–70. doi: 10.1038/s41375-019-0530-9 (PMC8076004; doi:10.1038/s41375-019-0530-9)
Supplement: Supplementary file 2 — Supplementary Figure 1A [file 41375_2019_530_MOESM2_ESM.pptx]

## Slide 1
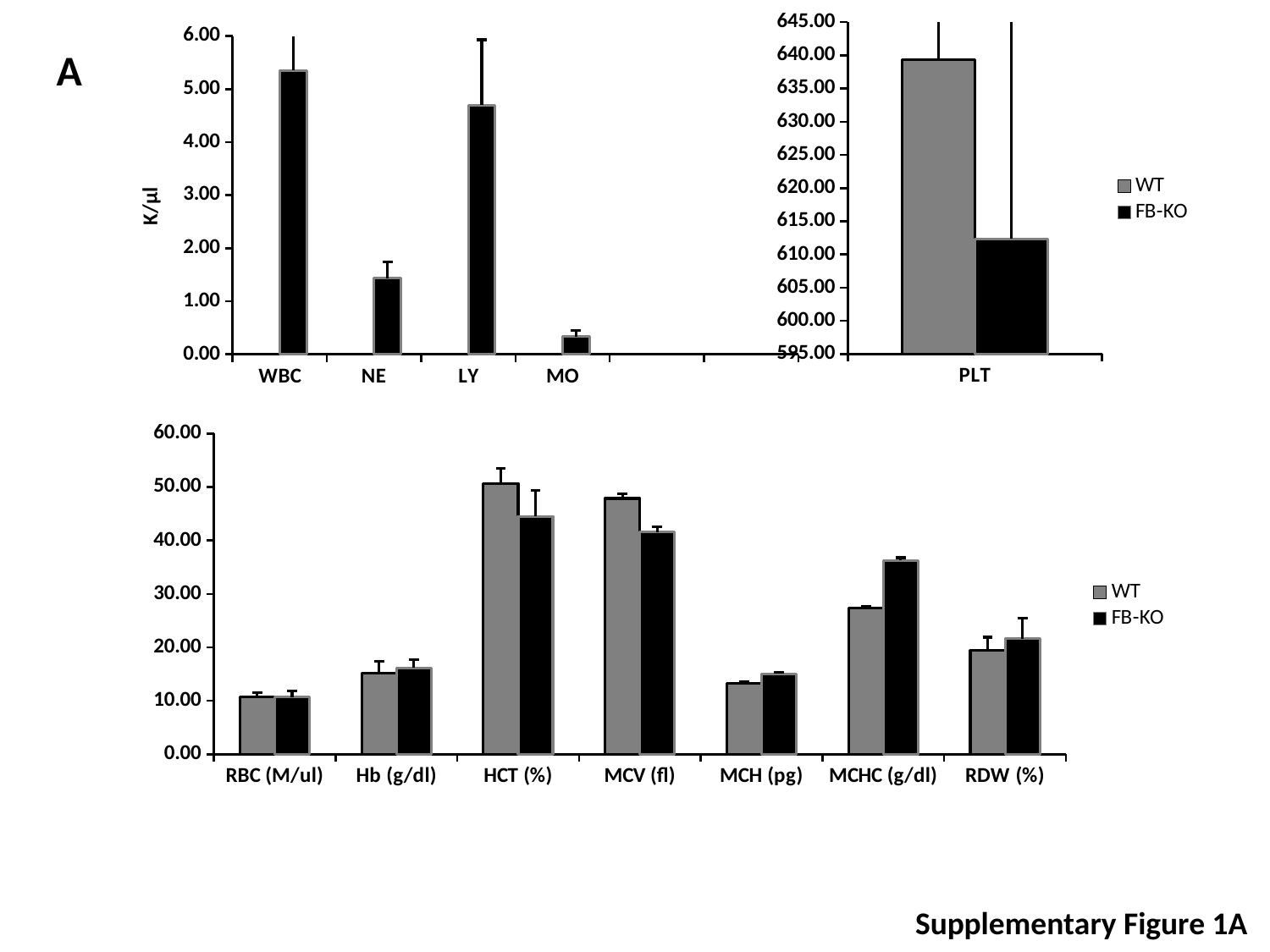

### Chart
| Category | WT | FB-KO |
|---|---|---|
| PLT | 639.3333333333334 | 612.3333333333334 |
### Chart
| Category | WT | FB-KO |
|---|---|---|
| WBC | 5.026666666666666 | 5.35 |
| NE | 1.9000000000000001 | 1.4333333333333333 |
| LY | 5.003333333333334 | 4.7 |
| MO | 0.5733333333333334 | 0.3333333333333333 |A
### Chart
| Category | WT | FB-KO |
|---|---|---|
| RBC (M/ul) | 10.76 | 10.700000000000001 |
| Hb (g/dl) | 15.200000000000001 | 16.133333333333333 |
| HCT (%) | 50.699999999999996 | 44.53333333333334 |
| MCV (fl) | 47.866666666666674 | 41.6 |
| MCH (pg) | 13.2 | 15.066666666666668 |
| MCHC (g/dl) | 27.35 | 36.233333333333334 |
| RDW (%) | 19.400000000000002 | 21.600000000000005 | Supplementary Figure 1A
