# Supplementary material for: Novel evidence that an alternative complement cascade pathway is involved in optimal mobilization of hematopoietic stem/progenitor cells in Nlrp3 inflammasome-dependent manner
Source: Leukemia. 2019 Jul 26;33(12):2967–70. doi: 10.1038/s41375-019-0530-9 (PMC8076004; doi:10.1038/s41375-019-0530-9)
Supplement: Supplementary file 3 — Supplementary Figure 1 B and C [file 41375_2019_530_MOESM3_ESM.pptx]

## Slide 1
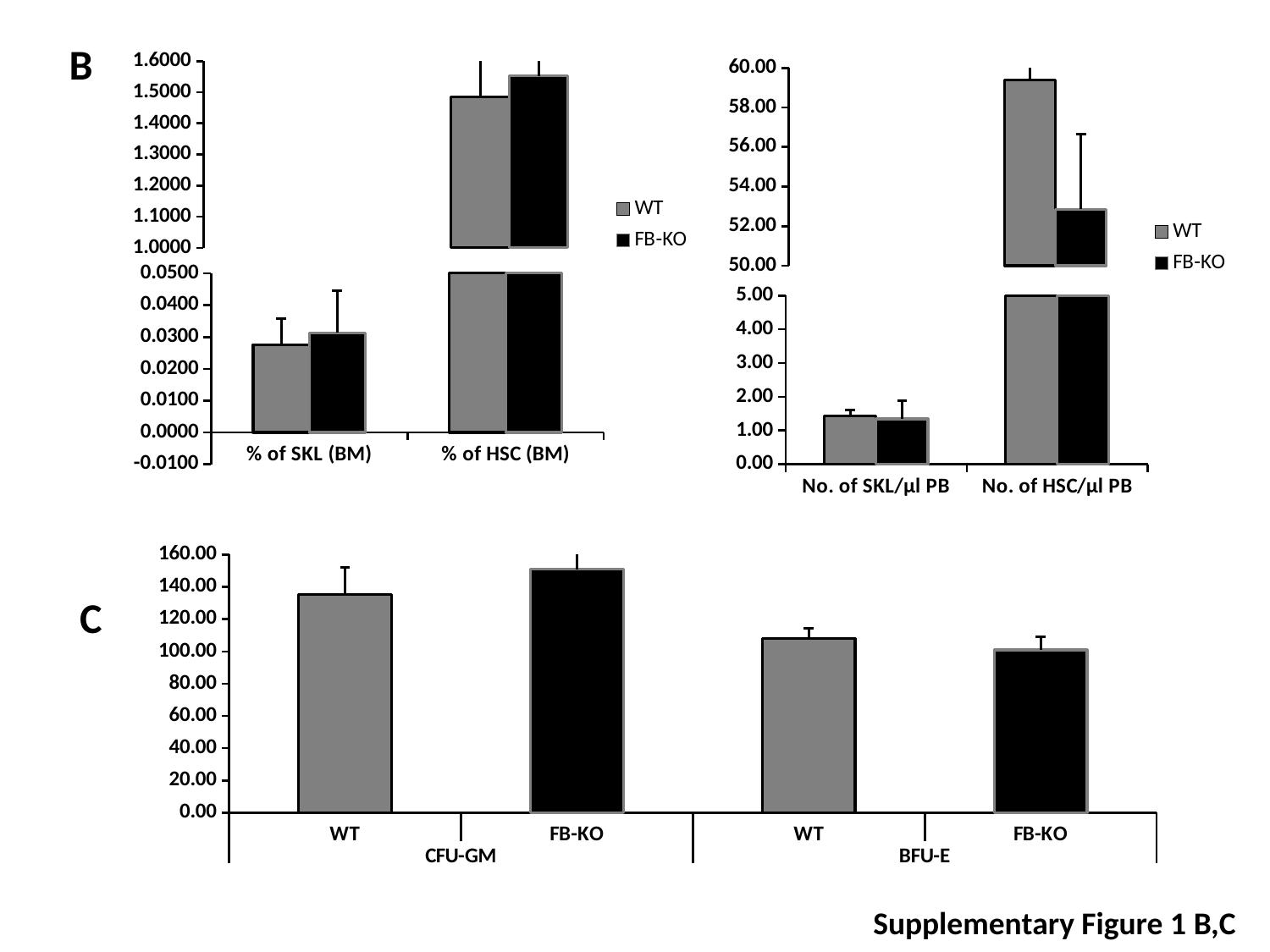

B
### Chart
| Category | WT | FB-KO |
|---|---|---|
| % of SKL (BM) | 0.027612613340792874 | 0.031307791900372056 |
| % of HSC (BM) | 1.4853681440115247 | 1.552659922279727 |
### Chart
| Category | WT | FB-KO |
|---|---|---|
| % of SKL (BM) | 0.027612613340792874 | 0.031307791900372056 |
| % of HSC (BM) | 1.4853681440115247 | 1.552659922279727 |
### Chart
| Category | WT | FB-KO |
|---|---|---|
| No. of SKL/µL PB | 1.4330978613147618 | 1.346482468794349 |
| No. of HSC/µL PB | 59.40660969861034 | 52.844970346879236 |
### Chart
| Category | WT | FB-KO |
|---|---|---|
| No. of SKL/µl PB | 1.4330978613147618 | 1.346482468794349 |
| No. of HSC/µl PB | 59.40660969861034 | 52.844970346879236 |
### Chart
| Category | |
|---|---|
| WT | 135.1375 |
| FB-KO | 150.93125 |
| WT | 107.82499999999999 |
| FB-KO | 100.9375 |C
 Supplementary Figure 1 B,C
