# Supplementary material for: Novel evidence that an alternative complement cascade pathway is involved in optimal mobilization of hematopoietic stem/progenitor cells in Nlrp3 inflammasome-dependent manner
Source: Leukemia. 2019 Jul 26;33(12):2967–70. doi: 10.1038/s41375-019-0530-9 (PMC8076004; doi:10.1038/s41375-019-0530-9)
Supplement: Supplementary file 4 — Supplementary Figure 2A [file 41375_2019_530_MOESM4_ESM.pptx]

## Slide 1
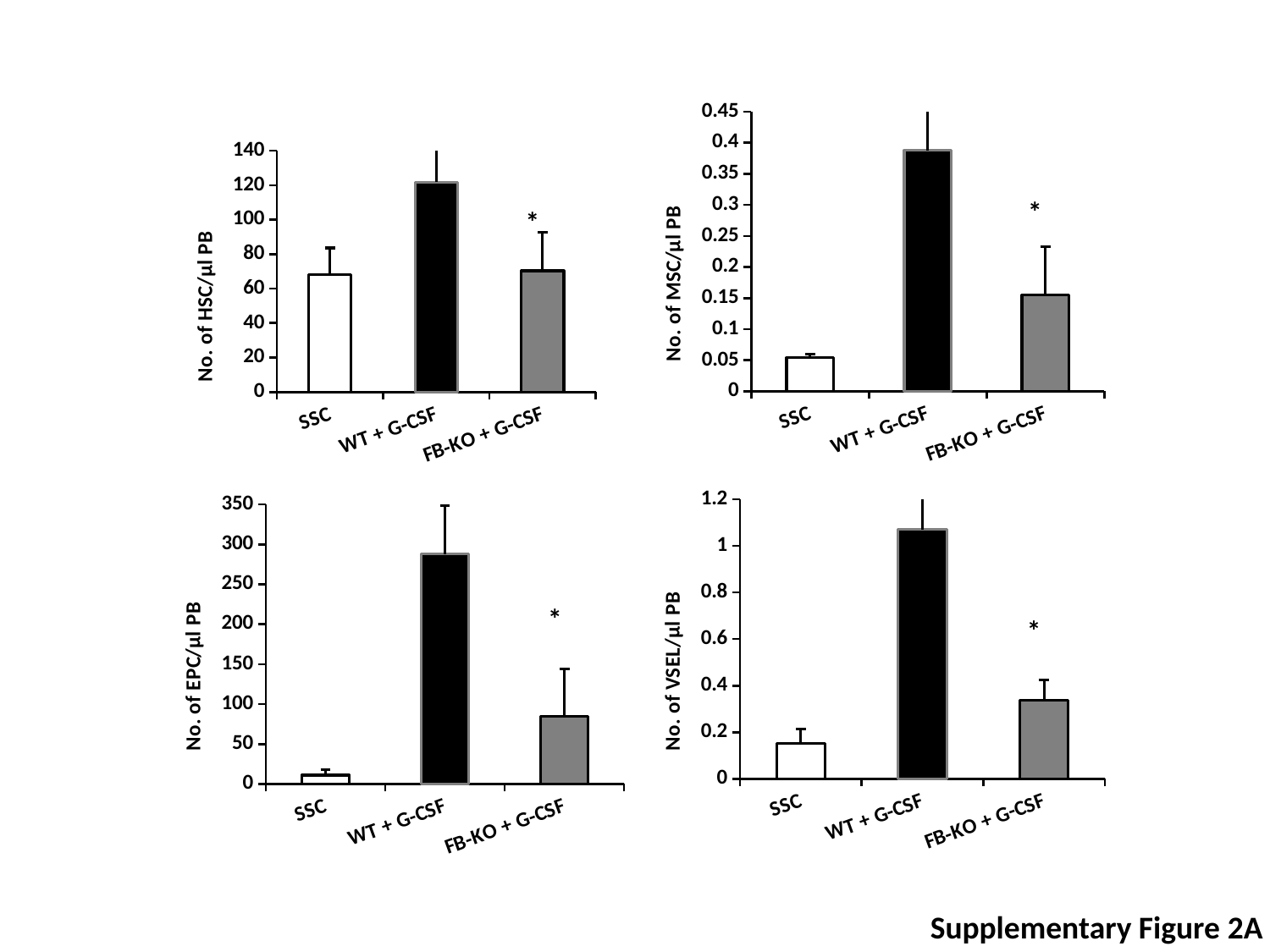

### Chart
| Category | |
|---|---|
| SSC | 0.054 |
| WT + G-CSF | 0.38781906627739643 |
| FB-KO + G-CSF | 0.1546371493312084 |*
### Chart
| Category | |
|---|---|
| SSC | 68.02 |
| WT + G-CSF | 121.72832588006183 |
| FB-KO + G-CSF | 70.38183353588498 |*
### Chart
| Category | |
|---|---|
| SSC | 0.1512 |
| WT + G-CSF | 1.0709838504779925 |
| FB-KO + G-CSF | 0.3375796345918635 |*
### Chart
| Category | |
|---|---|
| SSC | 11.008799999999999 |
| WT + G-CSF | 287.85296244789333 |
| FB-KO + G-CSF | 84.97773629825966 |*
 Supplementary Figure 2A
