# Supplementary material for: Novel evidence that an alternative complement cascade pathway is involved in optimal mobilization of hematopoietic stem/progenitor cells in Nlrp3 inflammasome-dependent manner
Source: Leukemia. 2019 Jul 26;33(12):2967–70. doi: 10.1038/s41375-019-0530-9 (PMC8076004; doi:10.1038/s41375-019-0530-9)
Supplement: Supplementary file 5 — Supplementary Figure 2B [file 41375_2019_530_MOESM5_ESM.pptx]

## Slide 1
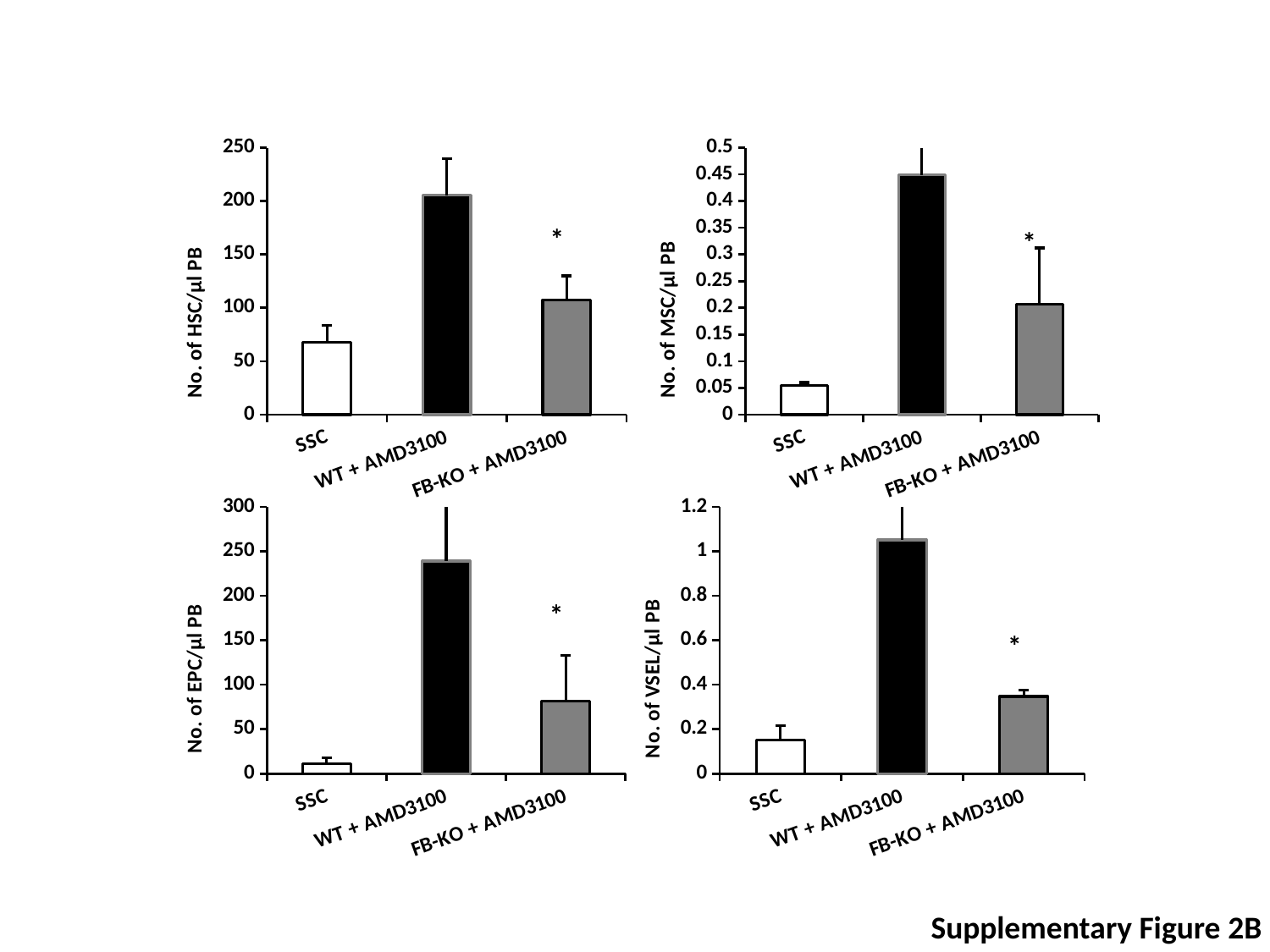

### Chart
| Category | |
|---|---|
| SSC | 68.02 |
| WT + AMD3100 | 205.54638666666668 |
| FB-KO + AMD3100 | 107.51648 |*
### Chart
| Category | |
|---|---|
| SSC | 0.054 |
| WT + AMD3100 | 0.44894000000000006 |
| FB-KO + AMD3100 | 0.20631999999999998 |*
### Chart
| Category | |
|---|---|
| SSC | 11.008799999999999 |
| WT + AMD3100 | 238.99607999999998 |
| FB-KO + AMD3100 | 81.3445 |*
### Chart
| Category | |
|---|---|
| SSC | 0.1512 |
| WT + AMD3100 | 1.0512266666666668 |
| FB-KO + AMD3100 | 0.3469466666666667 |*
 Supplementary Figure 2B
